# Autoimmune-associated hemophagocytic syndrome complicated by anti-MDA5 antibody-positive dermatomyositis successfully treated with immunosuppressive therapy: A case report

**DOI:** 10.1097/MD.0000000000043229

**Published:** 2025-07-04

**Authors:** Riko Kamada, Makoto Hibino, Hikari Higa, Shigehiro Watanabe, Kazunari Maeda, Noriyoshi Ishikawa, Takuya Kakutani, Tetsuri Kondo

**Affiliations:** a Department of Respiratory Medicine, Shonan Fujisawa Tokushukai Hospital, Fujisawa 251-0041, Kanagawa, Japan; b Division of Rheumatology, Shonan Kamakura General Hospital, Kamakura 247-8533, Kanagawa, Japan; c Shonan Oiso Hospital, Oiso 259-0114 Kanagawa, Japan; d Department of Pathology, Shonan Fujisawa Tokushukai Hospital, Fujisawa 251-0041, Kanagawa, Japan.

**Keywords:** anti-MDA5 antibody-positive dermatomyositis, hemophagocytic syndrome, hyperferritinemia, liver dysfunction, rapidly progressive interstitial pneumonia

## Abstract

**Rationale:**

Anti-melanoma differentiation–associated gene 5 (anti-MDA5) antibody-positive dermatomyositis is frequently complicated by rapidly progressive interstitial lung disease and may present with life-threatening hemophagocytic syndrome, a critical but often under-recognized complication. Prompt diagnosis and intervention are essential to improve patient outcomes.

**Patient concerns:**

A 38-year-old Japanese woman presented with fever, arthralgia, erythema, and elevated liver enzymes. Physical examination revealed chilblain-like erythema, Gottron and reverse Gottron signs, and tenderness in the thighs.

**Diagnoses:**

Blood tests revealed pancytopenia and elevated soluble interleukin-2 receptor and ferritin levels. Bone marrow aspiration showed macrophage activation and hemophagocytosis. Imaging revealed ground-glass opacities in both lower lung lobes and bilateral thigh myositis. The presence of anti-MDA5 antibodies led to the diagnosis of anti-MDA5-positive dermatomyositis complicated by autoimmune-related hemophagocytic syndrome. Liver biopsy suggested cytokine storm-mediated hepatocyte damage and macrophage infiltration.

**Interventions:**

The patient received steroid pulse therapy, tacrolimus, and intravenous cyclophosphamide.

**Outcomes:**

Pancytopenia and liver dysfunction improved significantly following treatment. The patient was transitioned to outpatient care. After 4 years of follow-up, during which she received 6 courses of cyclophosphamide, steroids, and tacrolimus were successfully tapered off, and she remained in remission without recurrence.

**Lessons:**

This case underscores the importance of early recognition of hemophagocytic syndrome in patients with anti-MDA5-positive dermatomyositis. Prompt bone marrow evaluation and aggressive immunosuppressive therapy may lead to favorable long-term outcomes. Further studies are warranted to establish standardized treatment strategies for autoimmune-related hemophagocytic syndrome.

## 1. Introduction

Dermatomyositis (DM) is an idiopathic inflammatory myopathy that affects the skin, skeletal muscles, lungs, and other organs, resulting in various systemic manifestations.^[1]^ Clinically amyopathic dermatomyositis (CADM) is a distinct subtype of DM characterized by typical DM cutaneous findings but with minimal or no evidence of myositis.^[2]^ Anti-melanoma differentiation-associated gene 5 (MDA5) antibodies were originally identified to be associated with CADM in combination with rapidly progressive interstitial lung disease (RP-ILD). In East Asia, anti-MDA5 antibody-positive DM is frequently associated with RP-ILD and high mortality rates.^[3]^

Hemophagocytic syndrome (HPS) is a life-threatening syndrome characterized by clinical signs and symptoms of intense immune activation, such as fever, cytopenia, hepatosplenomegaly, and hyperferritinemia.^[4]^ HPS is classified into primary and secondary forms. Infections, autoinflammatory and autoimmune diseases, malignancies, and acquired immune deficiency syndrome can cause secondary HPS.^[5]^ HPS secondary to rheumatic diseases is also known as macrophage activation syndrome (MAS). Adult-onset Still disease and systemic lupus erythematosus are sometimes complicated by HPS; however, DM is rare in autoimmune-associated HPS (AAHS), accounting for only 6.9% of all patients with AAHS. AAHS is potentially fatal, with a mortality rate of 12.9%.^[6]^

Herein, we report a case of anti-MDA5 antibody-positive DM with HPS that was successfully treated with immunosuppressive therapy.

## 2. Case presentation

A 38-year-old Japanese woman presented with fever (with a temperature of 39°C), erythema of the extremities, and arthralgia for 1 month. She had visited a dermatologist, and 1.5 mg betamethasone was started orally for 2 weeks, with a provisional diagnosis of erythema exudativum multiforme. One week after starting the treatment, the dose was reduced to 1 mg, and she was referred to our hospital for evaluation of elevated liver enzyme levels (aspartate aminotransferase, 658 U/L; alanine aminotransferase, 720 U/L). A liver biopsy performed at the Department of Hepatology showed no specific findings, and the underlying disease remained unidentified; hence, the patient was referred to the Department of General Internal Medicine for further evaluation.

The patient’s medical history revealed an ovarian cyst and appendicitis. She was not on any regular medication and had been taking folic acid supplements for 4 months. She had no history of smoking or excessive alcohol consumption. Her grandmother had rheumatoid arthritis. She reported no history of transfusion, overseas travel, or recent consumption of raw oysters or meat.

**Figure 1.**
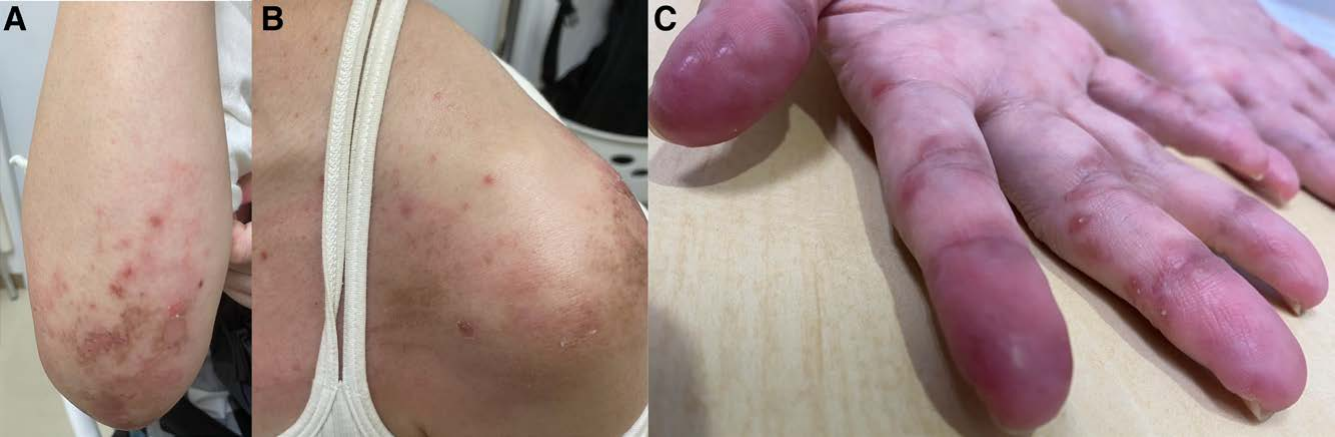
Gottron sign on the elbow (A) and shoulder (B), with reverse Gottron sign and chilblain-like erythema of the fingers (C).

Upon admission, physical examination revealed a body temperature of 37.9°C, blood pressure of 100/75 mmHg, pulse rate of 93/min, and percutaneous oxygen saturation of 98% (in room air). No ocular conjunctival anemia, ocular conjunctival yellowing, or cervical lymphadenopathy was observed. Her heart sounds were regular, with no murmurs, and respiratory sounds were clear. Her abdomen was flat and soft, with no tenderness. Tenderness and swelling of the wrist and knee joints were observed, but leg edema was absent. Myalgia was present in both thighs, but no muscle weakness was noted. Periungual erythema; chilblain-like erythema of the fingers; Gottron sign on the dorsum of the hands, shoulders, and elbows; and the inverse Gottron sign on the palms were observed (Fig. 1).

**Figure 2.**
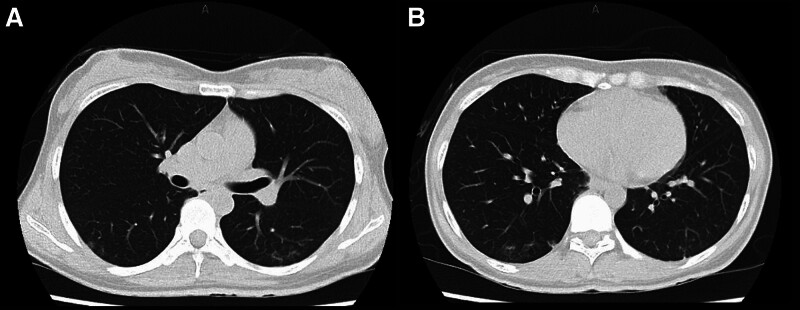
Computed tomography findings of the chest showing ground-glass opacity dorsally in both lower lung lobes. (A) At the level of segment 6 (S6). (B) At the lung bases.

**Figure 3.**
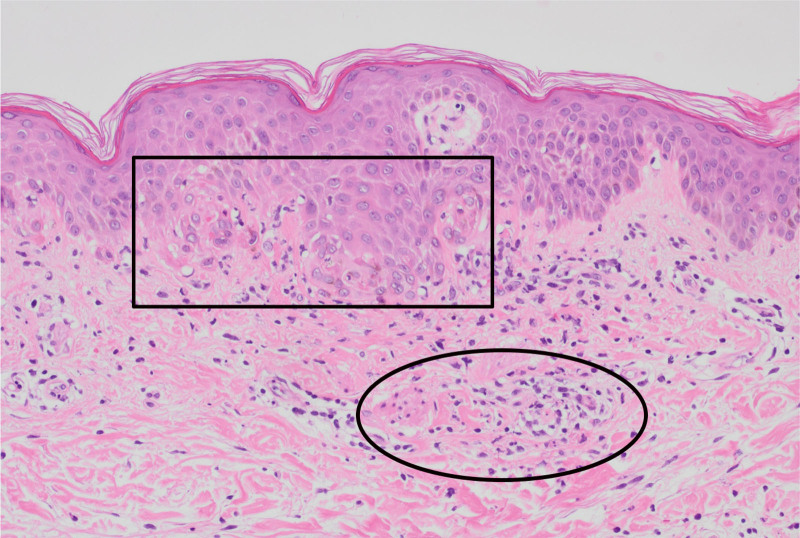
Degeneration of the basement membrane with a liquefactive pattern (square) and perivascular inflammatory cell infiltration in the dermis (circle) were noted.

Laboratory findings on admission are shown in Table 1. The patient had pancytopenia, liver dysfunction, and elevated ferritin and soluble interleukin-2 receptor (sIL-2R) levels. The creatine phosphokinase level was within the reference range; however, aldolase and myoglobin levels were elevated. There was no evidence of acute-phase infection with the presence of viral antibodies for hepatitis, Epstein–Barr virus, cytomegalovirus, or parvovirus. The result of severe acute respiratory syndrome coronavirus 2 nasopharynx loop-mediated isothermal amplification was negative. Chest computed tomography revealed dorsal ground-glass opacities in both lower lung lobes (Fig. 2), suggesting the presence of ILD. The serum levels of Krebs von den Lungen-6 were elevated (730 U/mL). Blood cultures were negative, and abdominal ultrasound showed mild splenomegaly. Magnetic resonance imaging, diffusion-weighted imaging with background suppression, and short-tau inversion recovery revealed hyperintensity in both quadriceps femoris muscles, suggesting myositis (Fig. 3).

**Table 1:** Laboratory data on admission.

WBC (/μL)	1900	TP (mg/dL)	6.1	ANA (folds)	1:40
Neutrophils (%)	57.6	Albumin (mg/dL)	2.5	Homogenous	40
Lymphocytes (%)	30.4	T-Bil (mg/dL)	0.5	Speckled	40
Monocytes (%)	11	AST (U/L)	592	Anti-SS-A/Ro (U/mL)	<0.5
Eosinophils (%)	1	ALT (U/L)	402	MPO-ANCA (IU/mL)	<0.5
Basophils (%)	0	LDH (U/L)	367	PR3-ANCA (IU/mL)	<0.5
Hb (g/dL)	11.1	ALP (U/L)	327	Anti-ARS (INDEX)	<5
Plt (×10^4^/μL)	13.3	γGTP (U/L)	56	Anti-MDA5 (INDEX)	2710
–	–	BUN (mg/dL)	10.7	EBV VCA-IgG (folds)	320
PT-INR	1.07	Creatinine (mg/dL)	0.46	EBV VCA-IgM (folds)	<10
APTT (s)	42.4	Na (mEq/L)	138	EBNA (folds)	160
Fib (mg/dL)	276	K (mEq/L)	3.9	CMV-IgG (AU/mL)	<6
FDP (μg/mL)	7.1	Cl (mEq/L)	104	CMV-IgM (S/CO)	<0.85
D-Dimer (μg/mL)	2.82	Ca (mg/dL)	8.5	Parvovirus B19 IgM (INDEX)	0.82
–	–	CRP (mg/dL)	1	HA-IgM (S/CO)	0.1
–	–	TG (mg/dL)	146	HBs antigen (IU/mL)	0
–	–	Ferritin (ng/mL)	1452	HBs antibody (mIU/mL)	0
–	–	sIL-2R (U/mL)	1067	HBc antibody (S/CO)	0.2
–	–	KL-6 (U/mL)	730	HCV antibody (S/CO)	0.04
–	–	CPK (U/L)	138	HEV-IgG (folds)	320
–	–	ALD (U/L)	26.9	HEV-IgM (folds)	<10
–	–	Myoglobin (ng/mL)	96.8	AMA (folds)	<20

ALD = aldosterone, ALP = alkaline phosphatase, ALT = alanine aminotransferase, AMA = anti-mitochondrial antibody, ANA = antinuclear antibody, Anti-ARS = anti-aminoacyl tRNA synthetase, Anti-MDA5 = anti-melanoma differentiation-associated gene 5, Anti-SS-A/Ro = anti-Sjögren’s syndrome-associated antigen A/Ro antibody, APTT = activated partial thromboplastin time, AST = aspartate aminotransferase, BUN = blood urea nitrogen, CMV = cytomegalovirus, CPK = creatine phosphokinase, CRP = C-reactive protein, EBNA = Epstein–Barr virus nuclear antigen, EBV = Epstein–Barr virus, FDP = fibrin degradation products, Fib = fibrinogen, Hb = hemoglobin, HCV = hepatitis C virus, HEV = hepatitis E virus, INR = international normalized ratio, KL-6 = Krebs von den Lungen-6, LDH = lactate dehydrogenase, MPO-ANCA = myeloperoxidase antineutrophil cytoplasmic antibody, Plt = platelets, PR3-ANCA = proteinase 3-cytoplasmic antineutrophil cytoplasmic antibody, PT = prothrombin time, sIL-2R = soluble interleukin-2 receptors, T-Bil = total bilirubin, TG = triglycerides, TP = total protein, WBC = white blood cells, γGTP = gamma-glutamyl transpeptidase.

**Figure 4.**
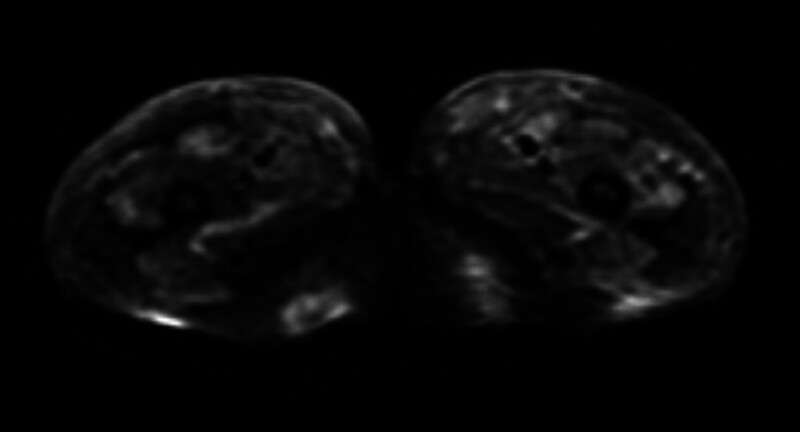
Hyperintense signals were observed in both quadriceps femoris muscles on magnetic resonance imaging, including diffusion-weighted imaging with background suppression and short-tau inversion recovery.

**Figure 5.**
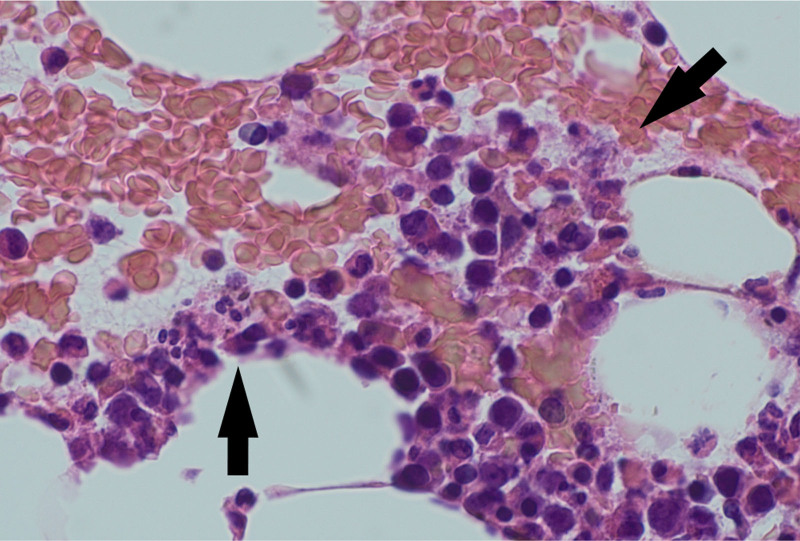
Pathological findings in the bone marrow revealed increased numbers of macrophages with hemophagocytosis (arrows) and no atypical cells.

A skin biopsy was performed for evaluating the keratinization of the erythematous right elbow. Liquid degeneration of the basement membrane and inflammatory cell infiltration around blood vessels in the dermis were observed, consistent with DM (Fig. 4). Bone marrow aspiration and biopsy showed increased numbers of macrophages demonstrating hemophagocytosis, without any malignancy (Fig. 5). Upon review of the liver biopsy pathology, histopathological findings showed spotty necrosis with lymphocyte and T-cell infiltration within hepatic lobules, minimal bile duct reaction, clusters of ceroid-laden macrophages, and Kupffer cells in variable clusters.

Tests for autoantibodies associated with connective tissue diseases revealed a markedly elevated anti-MDA5 antibody level of 2710 index (reference value < 32 index; anti-MDA5 enzyme-linked immunosorbent assay). All other antibodies were negative (Table 1). Based on myalgia in the thighs, elevated myogenic enzyme levels, myositis findings on magnetic resonance imaging, characteristic skin lesions, arthritis, fever, elevated C-reactive protein level, and positive anti-MDA5 antibodies, anti-MDA5 antibody-positive DM was diagnosed.

In addition to the diagnosis of DM, the patient fulfilled the general diagnostic criteria for MAS, which is commonly considered in autoimmune disease-associated HPS, and met the diagnostic criteria for AAHS proposed by Kumakura et al,^[7]^ which included pancytopenia, increased numbers of macrophages with hemophagocytosis in the bone marrow, underlying disease activity, and exclusion of other secondary causes of HPS. The possibility of HPS due to malignancy or certain infections, including viral and bacterial infections, was ruled out. Steroid pulse therapy (methylprednisolone 1000 mg for 3 days) and oral tacrolimus (initially 3 mg/day, increased to a maximum of 6 mg/day) were initiated, followed by administration of intravenous immunoglobulin (IVIG: 20 g/day for 5 consecutive days). After confirming an increase in blood cell counts, intravenous cyclophosphamide 500 mg (357 mg/m²) was administered monthly. After starting treatment, laboratory results showed improved blood cell, liver enzyme, and ferritin levels, and the ILD did not progress. The patient underwent 6 courses of cyclophosphamide therapy. The steroids were gradually tapered and discontinued after 3 years and 8 months of treatment, while tacrolimus was progressively reduced until cessation at the 4-year mark. Currently, 4 years and 10 months since the start of treatment, the serum MDA5 antibody levels have returned to normal, and the patient is being followed up in outpatient care without any evident recurrence.

## 3. Patient consent

Ethical approval was waived because the study is a case report that does not require ethical review under institutional guidelines.

Signed informed consent was obtained from the patient regarding the use of patient information for the purposes of writing a case report publication.

## 4. Discussion

We describe the case of a patient with anti-MDA5 antibody-positive DM complicated by MAS and AAHS, who initially presented to a dermatologist with a skin rash and high fever, followed by a hepatologist for elevated liver enzyme levels. Neither specialist initially recognized the underlying condition, and the cause of the abnormalities was ultimately determined to be manifestations of the diagnosed disease.

This case involves a patient with anti-MDA5 antibody-positive DM complicated by HPS, which is a form of MAS, who met the diagnostic criteria for AAHS proposed in Japan. Although genetic testing for primary HPS was not performed, it was suspected to be adult-onset, secondary HPS. AAHS was diagnosed based on the absence of findings suggestive of malignancy on bone marrow examination, along with imaging and other clinical assessments, negative serological tests for acute-phase viral infections, negative blood cultures excluding the possibility of systemic bacterial infection, and the presence of newly diagnosed active DM. The patient met 4 of the 8 diagnostic criteria for hemophagocytic lymphohistiocytosis as defined by the Histiocyte Society in 2004,^[8]^ but did not fulfill the 5 necessary criteria for a definitive diagnosis. The 4 met criteria were as follows: persistent high fever, splenomegaly, hemophagocytosis in the bone marrow, and a serum ferritin level ≥500 ng/mL. The criteria that were not met were as follows: pancytopenia (although present, the criteria for hemoglobin <9 g/dL, platelets < 100,000/µL, and neutrophils < 1000/µL were not met), hypertriglyceridemia or hypofibrinogenemia, low or absent natural killer cell activity (not measured in this case), and elevated sIL-2R level to > 2400 U/mL (not attained in this case, with a level of 1067 U/mL). This could be due to the initiation of systemic steroid therapy before the patient was fully evaluated at our hospital.

This case was referred for the evaluation of elevated liver enzyme levels, which can be primarily caused by 2 mechanisms in HPS^[9]^: cytokine storm-mediated hepatocyte damage and macrophage infiltration and hemophagocytosis in the liver. In this case, liver biopsy showed no obvious hemophagocytosis; however, ceroid-laden macrophages and Kupffer cells were observed, indicating macrophage infiltration. Furthermore, spotty necrosis with lymphocyte and T-cell infiltration was observed, further supporting cytokine storm as the cause of liver injury.

RP-ILD has a high fatality rate; however, this patient had mild ILD, which is rare in East Asia. Racial differences in the association between anti-MDA5 antibodies and RP-ILD have been described in previous cohorts. For instance, in Japanese and East Asian populations, ILD occurs in 82% to 100% of patients with anti-MDA5 antibody-positive DM, and RP-ILD is found in 39 to 100% of patients. The incidence of RP-ILD is lesser in Caucasian populations, with 38% to 73% of the patients with anti-MDA5 antibody-positive DM having ILD and 20% to 57% having RP-ILD.^[3]^ In East Asia, patients with anti-MDA5 antibody-positive DM often have RP-ILD and require at least triple therapy including glucocorticoids, cyclophosphamide, and tacrolimus at an early stage.

Patients with anti-MDA5 antibody-positive DM have elevated serum ferritin levels, which may be associated with the ILD activity in DM.^[10]^ Hyperferritinemia in rheumatic diseases may be suggestive of HPS but is difficult to detect in anti-MDA5 antibody-positive DM due to the association of hyperferritinemia with ILD activity and the rare coexistence of DM and HPS. In 12 cases of anti-MDA5 antibody-positive DM with HPS, Ding et al showed that the risk of HPS was greater when aspartate aminotransferase, lactate dehydrogenase, and ferritin levels were higher. Of the 12 cases, 11 were reported in East Asia. The median duration from DM onset to HPS development was 3 months, and the fatality rate was 50%.^[11]^ Based on the results of this study, HPS complications in patients with anti-MDA5 antibody-positive DM are presumed to be frequent in East Asia, similar to those in patients with RP-ILD. It is vital not to overlook concomitant HPS findings associated with hyperferritinemia.

Furthermore, in anti-MDA5 antibody-positive DM, ILD may progress rapidly, leading to severe respiratory failure, thereby rendering bone marrow aspiration and biopsy difficult even when HPS is suspected. Consequently, some cases of HPS may remain undiagnosed. Additionally, HPS may be overlooked when pancytopenia is attributed to other causes, such as immunosuppressive drug use or infections, rather than being recognized as part of AAHS. This is particularly relevant in RP-ILD, where potent immunosuppressants are frequently used, increasing both the risk of drug-induced cytopenia and susceptibility to infections. Given these challenges, a bone marrow examination should be performed whenever possible to determine the cause of hemopenia. A diagnosis of HPS is pivotal because it may require additional treatment.

Although no generally acceptable protocol for AAHS treatment has been established, controlling inflammation caused by the underlying disease is essential. The use of corticosteroids in combination with other immunosuppressive medications, such as cyclophosphamide, IVIG, and cyclosporine, is recommended.^[6]^ Intensive therapy could contribute to a better HPS prognosis.

This patient presented with fever, rash, and arthritis, along with mild ILD. Allenbach et al proposed 3 types of anti-MDA5 antibody-positive DM with different prognoses.^[12]^ One group (18.1%) corresponded to patients with rapidly progressive ILD and a very high mortality rate. The second group (55.4%) corresponded to patients with pure dermato-rheumatologic symptoms and a good prognosis. The third group (26.5%) corresponded to patients, mainly male, with severe skin vasculopathy, frequent signs of myositis, and an intermediate prognosis. Thus, anti-MDA5 antibody-positive DM presents with different phenotypes and symptoms, requiring collaboration across multiple departments. Our patient, a female with joint symptoms and mild ILD, is presumed to have the type 2 phenotype. Even phenotypes with good prognoses can be complicated by HPS.

In conclusion, anti-MDA5 antibody-positive DM is known to be associated with RP-ILD and may be complicated by HPS. Hyperferritinemia is associated with RP-ILD activity in anti-MDA5 antibody-positive DM; however, the possibility of HPS should also be considered. The diagnosis of HPS and timely therapeutic intervention may improve the prognosis of anti-MDA5 antibody-positive DM.

## Acknowledgments

We thank Editage (www.editage.jp) for English language editing, which enhanced the clarity and coherence of the manuscript.

## Author contributions

**Investigation:** Makoto Hibino.

**Writing – original draft:** Riko Kamada.

**Writing – review & editing:** Makoto Hibino, Hikari Higa, Shigehiro Watanabe, Kazunari Maeda, Noriyoshi Ishikawa, Takuya Kakutani, Tetsuri Kondo.

## References

[1] Sato S Hirakata M Kuwana M. Autoantibodies to a 140-kd polypeptide, CADM-140, in Japanese patients with clinically amyopathic dermatomyositis. Arthritis Rheum. 2005;52:1571–6. 15880816 10.1002/art.21023

[2] Sontheimer RD. Would a new name hasten the acceptance of amyopathic dermatomyositis (dermatomyositis siné myositis) as a distinctive subset within the idiopathic inflammatory dermatomyopathies spectrum of clinical illness? J Am Acad Dermatol. 2002;46:626–36. 11907524 10.1067/mjd.2002.120621

[3] Nombel A Fabien N Coutant F. Dermatomyositis with anti-MDA5 antibodies: bioclinical features, pathogenesis and emerging therapies. Front Immunol. 2021;12:773352. 34745149 10.3389/fimmu.2021.773352 PMC8564476

[4] Emile JF Abla O Fraitag S.; Histiocyte Society. Revised classification of histiocytoses and neoplasms of the macrophage-dendritic cell lineages. Blood. 2016;127:2672–81. 26966089 10.1182/blood-2016-01-690636 PMC5161007

[5] Janka GE Lehmberg K. Hemophagocytic syndromes-an update. Blood Rev. 2014;28:135–42. 24792320 10.1016/j.blre.2014.03.002

[6] Kumakura S Murakawa Y. Clinical characteristics and treatment outcomes of autoimmune-associated hemophagocytic syndrome in adults. Arthritis Rheumatol. 2014;66:2297–307. 24756912 10.1002/art.38672 PMC4271677

[7] Kumakura S Ishikura H Kondo M Murakawa Y Masuda J Kobayashi S. Autoimmune-associated hemophagocytic syndrome. Mod Rheumatol. 2004;14:205–15. 17143676 10.1007/s10165-004-0293-6

[8] Henter JI Horne A Aricó M. HLH-2004: diagnostic and therapeutic guidelines for hemophagocytic lymphohistiocytosis. Pediatr Blood Cancer. 2007;48:124–31. 16937360 10.1002/pbc.21039

[9] Dong J Xie F Jia L. Clinical characteristics of liver failure with hemophagocytic lymphohistiocytosis. Sci Rep. 2019;9:8125. 31148551 10.1038/s41598-019-43909-w PMC6544643

[10] Gono T Kawaguchi Y Ozeki E. Serum ferritin correlates with activity of anti-MDA5 antibody-associated acute interstitial lung disease as a complication of dermatomyositis. Mod Rheumatol. 2011;21:223–7. 21052763 10.1007/s10165-010-0371-x

[11] Ding Y Ge Y. Anti-melanoma differentiation-associated gene 5 antibody-positive dermatomyositis complicated with macrophage activation syndrome. Ther Adv Chronic Dis. 2022;13:20406223221098128. 35586303 10.1177/20406223221098128 PMC9109495

[12] Allenbach Y Uzunhan Y Toquet S.; French Myositis Network. Different phenotypes in dermatomyositis associated with anti-MDA5 antibody: study of 121 cases. Neurology. 2020;95:e70–8. 32487712 10.1212/WNL.0000000000009727 PMC7371381

